# Brain herniation into arachnoid granulations: an underrecognized posterior fossa finding

**DOI:** 10.1007/s00234-025-03692-0

**Published:** 2025-07-08

**Authors:** Gozde Ozer, Haci Ibrahim Ekinci, Rasime Pelin Kavak

**Affiliations:** 1 Hacettepe University, Ankara, Turkey; 2 Etlik City Hospital, Ankara, Turkey

**Keywords:** Arachnoid granulation, Brain herniation, 3D-Drive, MRI

## Abstract

**Purpose:**

To evaluate the prevalence, imaging characteristics, and associated findings of brain herniation into arachnoid granulations (BHAG) using the 3D driven equilibrium (DRIVE) sequence on a 3T MRI system.

**Materials and methods:**

This retrospective study included 695 patients who underwent temporal MRI between January and May 2024. Imaging findings were reviewed by two experienced radiologists. BHAG prevalence, locations, associated parenchymal damage (gliosis/encephalomalacia), and detectability with conventional MRI sequences were assessed. Demographic data and clinical information were also recorded.

**Results:**

BHAG was identified in 7.3% of cases (51/695) in the posterior fossa. Patients with BHAG were older than those without (p=0.003), with no gender preference (p=0.259). Cerebellar herniations were most frequent (47.1%), followed by temporal (45.1%) and occipital lobe herniations (17.6%). Parenchymal damage was observed in 25.5% of patients with BHAG. BHAG was undetectable with conventional MRI sequences in 40% of cases.

**Conclusion:**

BHAG is a common incidental finding in brain MRI and presumably not associated with any symptoms. 3D-DRIVE sequence has a higher sensitivity, particularly with the high-field imaging systems, in the detection of BHAG.

## Introduction

Arachnoid granulations (AGs) are invaginations of the arachnoid membrane into the dural venous sinuses and, less commonly, into the calvarium. They develop as a result of cerebrospinal fluid pulsations in areas of weak or defective dura, often at venous convergence points, drawing adjacent pia mater from the brain surface toward the AG. This same pulsatile force can also cause brain parenchyma to herniate into arachnoid granulations, a phenomenon known as brain herniation into arachnoid granulations (BHAG) [1].

BHAG has recently been described as an imaging finding with elusive etiology [2]. Although it is usually incidental finding on brain MRI, some studies have suggested association between BHAG and symptoms such as dizziness, headache, and pulsatile tinnitus, particularly in the context of increased intracranial pressure [3–5]. Conversely, the frequent presence of BHAG in asymptomatic individuals has raised the possibility of spontaneous development [6]. Furthermore, tissue damage, including atrophy and gliosis, has been reported in both the herniated tissue and adjacent parenchyma [7].

With advancements in MRI spatial resolution, particularly with the advent of high-field imaging systems, it is not surprising that the detection of BHAG may increase over time. Recent studies suggest that BHAG remains underrecognized by radiologists [6, 8]. This study aims to evaluate the prevalence and associated imaging findings of BHAG using the 3D- driven equilibrium (DRIVE) sequence on a 3 T MRI system.

## Methods

### Patient selection

Ethical approval was obtained from the institutional review board (AESH-BADEK-2024-791) and written informed consent was waived since this study was a retrospective. The images of 704 patients who underwent temporal MRI in our hospital between January 2024 and May 2024 were evaluated. Nine patients with artifacts obscuring parts of the intracranial cavity, marked motion artifacts, or inadequate imaging field of view were excluded. And a total of 695 patients were included in the study. Demographic data, clinical information for MRI, and presence of history of idiopathic intracranial hypertension (IIH) were recorded.

## Imaging technique

MRIs were performed with a Philips Ingenia 3 T Elition X scanner (Philips Healthcare, Best, Netherlands) using standard head coils. Standard MRI protocols included axial and coronal T1-weighted and T2-weighted sequences, diffusion weighted imaging, and 3D-DRIVE sequence posterior fossa. Additionally, all MRI studies included axial T2 weighted sequence of brain. The imaging parameters of temporal MRI are summarized in Table 1.

**Table 1:** MR sequence parameters for Temporal MRI

Sequence	Plane	TR (ms)	TE (ms)	Slice thickness (mm)	Fat saturation	Matrix	FOV (mm)
3D-DRIVE	Axial	2000	235	1	-	360 × 287	180 × 180
T1 SE	Coronal	450	13	3	-	180 × 149	180 × 180
T1 SE	Axial	501	13	3	-	256 × 212	180 × 180
T2 TSE	Axial	3000	80	5	-	288 × 209	230 × 176
DWI	Coronal	2000	59	3	+	100 × 90	180 × 180
T1 SE C+	Coronal	450	13	3	-	180 × 149	180 × 180
T1 SE C+	Axial	607	13	3	+	256 × 212	180 × 180

*3D-DRIVE* 3D-driven equilibrium, *DWI* diffusion weighted imaging, *FOV* field of view, *SE* spin echo, *TSE* turbo spin echo

## Image analysis

Patients’ images were evaluated on the PACS by two radiologists with 4 and 12 years of experience, respectively. Any discrepancies in interpretation were resolved by consensus. For each case, radiologists assessed the following: the presence and location of arachnoid granulations (AG); the presence and extent of brain or cerebellar herniation into the AGs; the parenchymal origin of the BHAG; the site of herniation into the dural venous sinus; evidence of encephalomalacia or atrophy in the adjacent parenchyma; and signal alterations (e.g., gliosis) in the herniated or surrounding brain tissue.

### Statistical analysis

Statistical analyses were performed using IBM SPSS Statistics for Windows, Version 22.0 (Armonk, NY: IBM Corp; 2013). The normality of age distribution was evaluated using the Kolmogorov-Smirnov and Shapiro-Wilk tests. Descriptive analyses were presented as mean (± standard deviation) for normally distributed data and median (minimum-maximum) for non-normally distributed data. Difference between groups in the cross-tables was compared using the Chi-square. The Mann-Whitney U test was used to test the differences in median age between patients in the groups since the age data were not normally distributed. The Chi-square, Fisher’s exact and Yates continuity correction tests were performed to evaluate the association between symptoms and BHAG presence. A p value of less than 0.05 was considered statistically significant.

## Results

A total of 695 patients’ (328 female, 367 male) temporal MRIs were evaluated. The median age for all patients is 52 (min-max = 20–85). Seventy-three brain/cerebellum herniations was detected in 51 patients (mean age = 57 years, min-max = 27–84; 54.9% female (*n* = 28), 45.1% male (*n* = 23), corresponding to 7.3% of all cases. There was no gender preference (*p* = 0.259). Patients with BHAG were older than those without (*p* = 0.003).

Hearing loss was the most common indication for imaging in 20 patients (39.2%) with BHAG. This was followed by tinnitus (*n* = 9, 17.6%), cholesteatoma (*n* = 9, 17.6%), vertigo (*n* = 3, 5.9%), chronic otitis media (*n* = 3, 5.9%), cerebellopontine angle tumors (*n* = 3, 5.9%), and facial paralysis (*n* = 2, 3.9%). IIH was not confirmed in any patient according to medical records. Additionally, the most common indications in patients without BHAG were hearing loss (*n* = 175), cholesteatoma (*n* = 121), and tinnitus (*n* = 80). There was no difference in the presence of BHAG between those with and without hearing loss (*p* = 0.093), those with and without tinnitus (*p* = 0.391), and those with and without cholesteatoma (*p* = 0.988).

A total of 51 patients were analyzed, 27 (52.9%) had no accompanying AGs, 12 patients (23.5%) had a single AG, and 12 patients (23.5%) had multiple AGs.

**Fig. 1:**
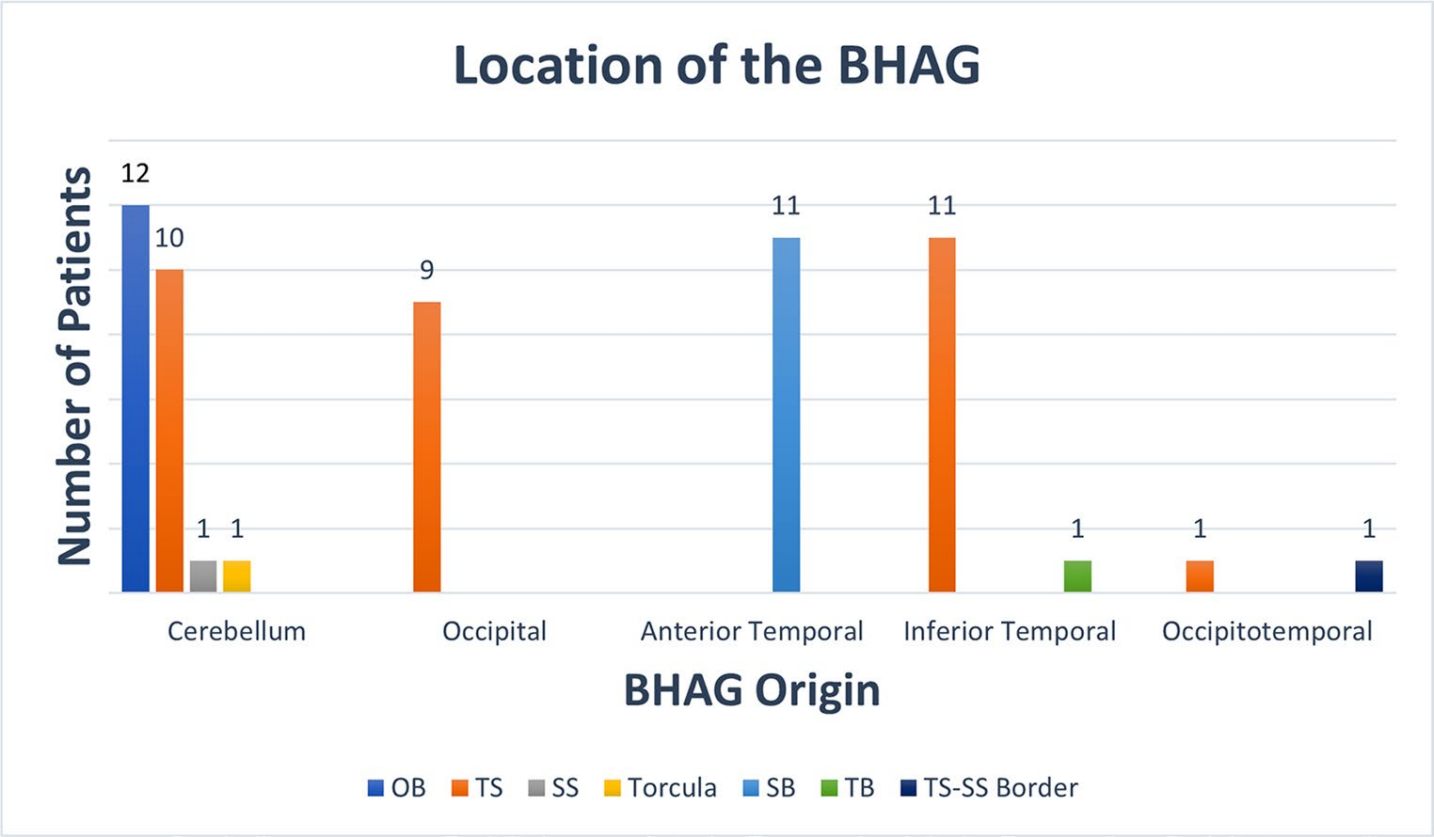
The diagram shows that location of the herniations. (OB: occipital bone, TS: transverse sinus, SS: sigmoid sinus, SB: sphenoid bone, TB: temporal bone, TS-SS Border: transverse sinus-sigmoid sinus border)

Cerebellar herniations were observed in 24 patients (47.1%), occipital lobe herniations in 9 patients (17.6%), occipitotemporal lobe herniations in 2 patients (3.9%), and temporal herniations in 23 patients (45.1%). Of these, 6 patients (12%) with cerebellar herniations had bilateral presentation, one patient (2%) with occipital lobe herniation had bilateral presentation, and 5 patients (9.8%) with temporal herniations had bilateral presentation. Among the 51 patients with identified herniations, 12 patients (23.5%) had more than one BHAG. The locations of the herniations are summarized visually in Fig. 1.

**Fig. 2:**
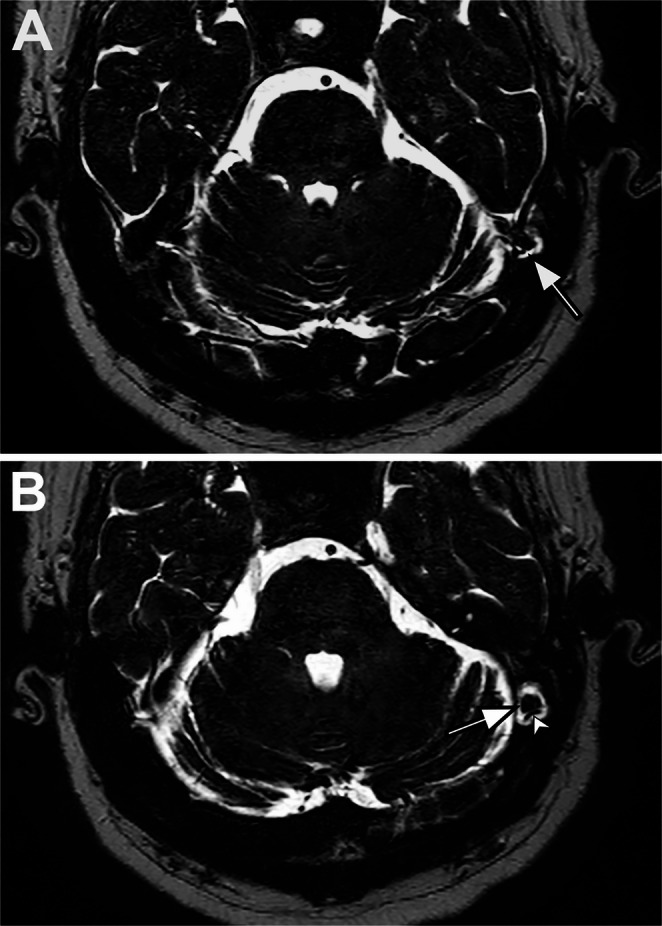
Fifty-nine-year-old male with hearing loss. **A** Axial 3D-DRIVE image shows the herniation of inferior temporal lobe into transverse sinus (arrow). **B** Note that vascular loop (arrowhead) in arachnoid granulation adjacent to the herniated parenchyma

**Fig. 3:**
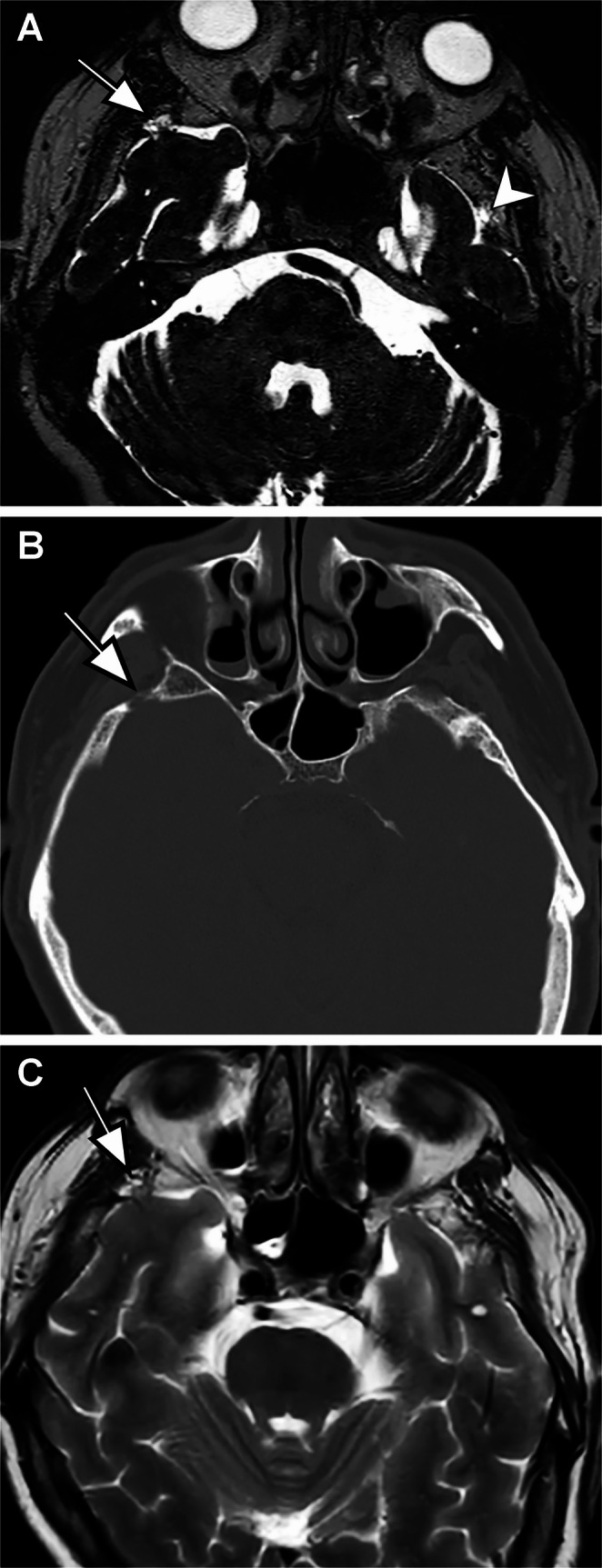
Forty-nine-year-old male with tinnitus. **A** Axial 3D-DRIVE image demonstrates that right anterior temporal lobe herniation (arrow) and left anterior temporal pit without herniation (arrowhead). **B** Axial head CT image (bone window) shows bone defect (arrow) at the level of herniation. **C** Note that herniation (arrow) is barely discernible on the axial T2-weighted MR image

Cerebellar herniations (24 patients) were most frequently localized to the occipital bone (*n* = 12, 50%), followed by the transverse sinus (*n* = 10, 41.7%), with single case involving the sigmoid sinus (*n* = 1, 4.2%) and torcula (*n* = 1, 4.2%). Occipital lobe herniations (*n* = 9) were all localized to the same-sided transverse sinus. Inferior temporal lobe herniations (*n* = 12) were predominantly localized to the transverse sinus (*n* = 11, 91.7%) (Fig. 2), with one case localized to the temporal bone (*n* = 1, 8.3%). Occipitotemporal lobe herniations (*n* = 2) were equally localized to the transverse sinus-sigmoid sinus border (*n* = 1, 50%) and the transverse sinus (*n* = 1, 50%). Anterior temporal pole herniations (*n* = 11) were all localized to the sphenoid bone (Fig. 3).

**Fig. 4:**
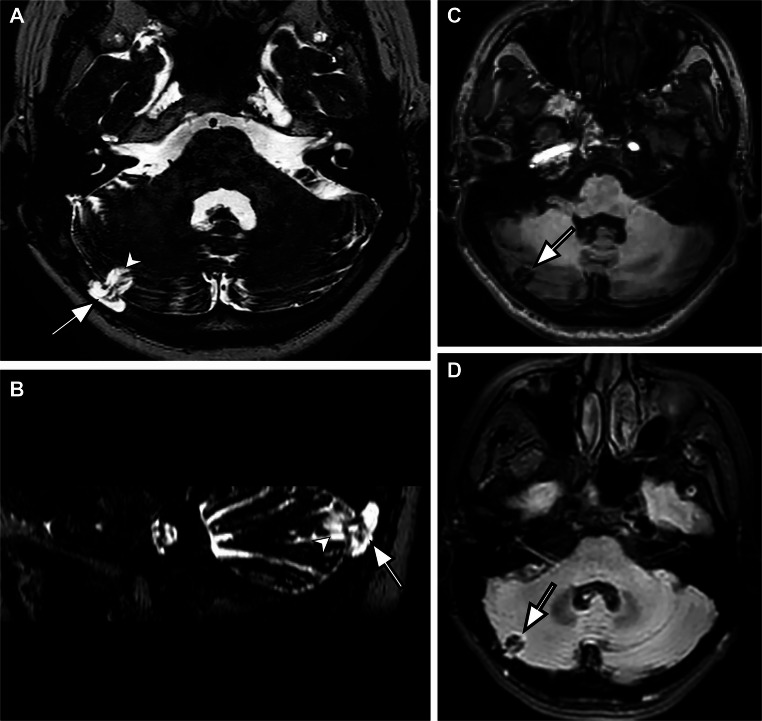
Sixty-five-year-old female patient with vertigo underwent temporal and brain MRI. **A** Axial and (**B**) sagittal reformatted 3D-DRIVE images show cerebellar herniation into giant arachnoid granulation (arrow) and encephalomalacia in adjacent parenchyma (arrowhead). **C** Axial T1-weighted MR image and (**D**) axial FLAIR image demonstrate parenchymal encephalomalacia and gliosis in both herniated and adjacent parenchyma

Among the 51 patients, 13 (25.5%) had parenchymal damage in herniated parenchyma. Six patients had gliosis only, 5 patients had encephalomalacia only, and 2 patients had both gliosis and encephalomalacia (Fig. 4).

When the detectability of herniations identified with the 3D-DRIVE sequence in both the posterior fossa and adjacent temporal and occipital regions was evaluated using conventional MRI sequences, herniations were detectable in 31 of 51 patients (60.8%), while they were not detectable in 20 patients (39.2%).

## Discussion

Our study demonstrated that BHAG is more common around the posterior fossa, with a prevalence of 7.3%, compared to previous studies. BHAG is presumably an underrecognized entity in radiology practice. Several hypotheses were suggested that possible pathogenesis of BHAG: weakness in dural layer of dural venous sinuses adjacent to protruding veins and arachnoid granulations, transient intracranial pressure changes, and/or spontaneously [6]. Understanding the potential mechanisms underlying BHAG is critical, as its prevalence and demographic characteristics provide further insights into its clinical significance. It has been reported that the frequency of BHAG increases with age and is more common in women than in men [1]. On contrary, Sade et al. showed that BHAG can be present in pediatric patients as frequently as adults [9]. Consistent with the first study, patients with BHAG in our study were older than those without. However, there was no difference between genders.

We found the higher prevalence of BHAG compared to previous literature, which can be attributed to several factors. First, we utilized the 3D-DRIVE sequence that provides an excellent contrast between cerebrospinal fluid and adjacent structures, superior anatomical detail of small structures due to high spatial resolution, and improved signal-to-noise and contrast-to-noise ratios, while minimizing magnetic susceptibility artifacts [10, 11]. Conventional brain MRI sequences can be insufficient for detection of BHAG probably because of the slice thickness. 3D volumetric T1 and T2 weighted images were found to be more accurate to detect BHAG [1, 9]. Consistent with that, %40 of BHAG could not be detected with conventional sequences in our study. Second, as mentioned above, we classified anterior temporal lobe herniations as BHAG in our study aligning with similar imaging and clinical features described in the literature.

Previous studies have suggested that BHAG may be associated with symptoms such as headache, tinnitus, and dizziness [5, 12, 13]. Smith et al. showed a strong association between IIH and BHAG [3]. However, many studies did not find a significant correlation between BHAG and clinical symptoms [14, 15]. Cavusoglu et al. reported the primary indications for temporal MRI as hearing loss (34%), tinnitus (27%), vertigo (31%), and peripheral facial paralysis (7%) [16]. In our study, temporal MRIs were retrospectively analyzed, and most patients presented with hearing loss and tinnitus, consistent with the literature. However, our study population consisted of patients with temporal bone/inner ear canal-related symptoms, and the lack of a control group limits our ability to draw conclusions about the direct relationship between BHAG and these symptoms. While our results align with previous studies suggesting that BHAG is often an incidental finding, the study design restricts the generalizability of these findings. The relationship between BHAG and clinical symptoms, particularly in the broader population, remains uncertain and warrants further investigation.

Middle cranial fossa pits and anterior temporal lobe herniations have generally been evaluated as an entity different from BHAG in literature. Anterior temporal lobe herniations are commonly named as “temporal meningoencephalocele”. Recent literature showed that occult anterior temporal herniations may be a cause of seizure in some patients [17, 18]. Otherwise, Benson et al. and Pettersson et al. showed that middle cranial fossa pits and anterior temporal herniations are more commonly an incidental finding and not related with the temporal lobe epilepsy [19, 20]. Also, parenchymal damage adjacent parenchyma was not found associated with seizures [20]. In our study, anterior temporal herniations were detected in 1% of patients, less than the literature [19]. None of the patients had seizures or a history of epilepsy according to medical records. Therefore, our data supported that anterior temporal herniations are probably incidental findings and should be classified as BHAG.

Our study has several limitations. First, it is a retrospective study, which inherently introduces potential biases. Second, our imaging protocol was anatomically limited to the posterior fossa and adjacent temporal and occipital regions, in line with our standard temporal MRI protocol. As such, supratentorial regions were not comprehensively assessed with volumetric imaging, which may have led to underdetection of BHAG in supratentorial areas. Our findings, therefore, apply specifically to the regions imaged, and future studies using whole-brain 3D T2-weighted sequences are warranted to fully evaluate BHAG prevalence and distribution. Third, the possible association between BHAG and various symptoms or diseases was investigated using clinical data from medical records, which may be insufficient. The inclusion of only temporal MRIs, the absence of a control group and the lack of patient evaluation for possible related symptoms limited our ability to draw certain conclusions about this association. Finally, while the 3D FLAIR sequence is the most useful for evaluating parenchymal damage, it was not included in our standard temporal MRI protocol, and only a few patients in our cohort underwent brain MRI with this sequence.

## Conclusions

BHAG is a common incidental finding on brain MRI and is presumably not associated with specific clinical symptoms. The 3D-DRIVE sequence, particularly when utilized with high-field imaging systems, demonstrates superior sensitivity in detecting BHAG, highlighting its value as a diagnostic tool in neuroradiological assessments.

## Data Availability

No datasets were generated or analysed during the current study.
